# Modified shehata surgery vs. laparoscopic One-stage orchiopexy for intra-abdominal cryptorchidism: a comparative retrospective study

**DOI:** 10.3389/fsurg.2026.1764561

**Published:** 2026-05-01

**Authors:** Shiyu Xiong, Xiuqiong Zhang, Yong Zeng, Feng Chen, Linshan Zeng, Wei Peng, Haijin Liu

**Affiliations:** 1 Department of Pediatrics Surgery, First Affiliated Hospital of Gannan Medical University, Ganzhou City, Jiangxi, China; 2 Department of Pediatrics, Dazhou City Integrated Traditional and Western Medicine Hospital (Dazhou Second People’s Hospital), Dazhou City, Sichuan, China; 3 Graduate School of Gannan Medical University, Ganzhou City, Jiangxi, China

**Keywords:** intra-abdominal cryptorchidism, laparoscopic surgery, pediatric urology, shehata surgery, staged laparoscopic orchiopexy, testicular volume

## Abstract

**Objective:**

To compare the therapeutic outcomes of a modified Shehata surgery with a condensed 4-week interstage interval vs. conventional laparoscopic one-stage orchiopexy for intra-abdominal cryptorchidism.

**Methods:**

We retrospectively analyzed 70 children with unilateral intra-abdominal cryptorchidism (July 2020–June 2022), allocated to modified Shehata (Group A, *n* = 35) or one-stage orchiopexy (Group B, *n* = 35). The modified procedure involved laparoscopic traction/fixation followed by second-stage orchiopexy at 4 weeks. Primary outcomes were testicular volume and blood flow (Doppler) assessed at 1, 3, and 6 months postoperatively. Secondary outcomes included serum testosterone (T), estradiol (E2), follicle-stimulating hormone (FSH) at 6 months, complication rates, success rate, and total treatment cost.

**Results:**

Baseline characteristics were comparable. Group A demonstrated significantly larger testicular volume at all postoperative timepoints (*P* < 0.05) and superior blood flow at 6 months (97.1% vs. 77.1% ‘Rich’ flow, *P* = 0.032). Regarding secondary outcomes, Group A had more favorable 6-month hormonal profiles (higher T, lower FSH/E2, *P* < 0.05), a lower surgery-related complication rate (0% vs. 17.14%, *P* = 0.033), and a higher success rate (100% vs. 82.86%, *P* = 0.033), but incurred higher total cost (*P* < 0.001).

**Conclusion:**

The modified Shehata surgery with a 4-week interval yields superior testicular development, perfusion, endocrine function, and lower complications compared to one-stage orchiopexy, albeit at higher cost.

## 1 Introduction

The optimal surgical management of intra-abdominal cryptorchidism remains a significant challenge in pediatric urology (1, 2). While orchiopexy is recommended within the first year of life to preserve testicular function and facilitate monitoring, the choice of technique for high-located testes remains contentious (3). One-stage laparoscopic orchiopexy is widely practiced but may be compromised by inadequate vascular length, leading to tension, testicular atrophy, or retraction (4). The Fowler-Stephens approach, which sacrifices the testicular vessels, carries a substantial risk of atrophy (5, 6). In response, Shehata et al. introduced a staged laparoscopic traction technique that promotes gradual vascular elongation, demonstrating superior outcomes in testicular perfusion and growth (7). However, the traditional 12-week inter-stage interval still leaves the testis exposed to the detrimental intra-abdominal thermal environment, which may compromise germ cell viability and also carries the risk of fixation suture slippage (8–10).

Our center previously introduced a modified Shehata procedure with a condensed 4-week interstage interval. Postoperative follow-up of up to 24 months has demonstrated favorable efficacy (11). However, from the perspectives of family financial burden, reduced anesthetic and surgical trauma, and holistic patient care, many families express a preference for the single-stage laparoscopic orchiopexy. To objectively evaluate and compare the advantages and disadvantages of these two surgical approaches for the treatment of intra-abdominal cryptorchidism, we conducted this retrospective comparative study (4, 12, 13).

## 2 Materials and methods

### 2.1 Study design and patient selection

This single-center, retrospective comparative study was conducted after obtaining approval from the Institutional Review Board of the First Affiliated Hospital of Gannan Medical University (Approval No. 022-22SC-2024). Clinical trial number: not applicable. The requirement for individual consent was waived due to the analysis's retrospective nature.

We reviewed the medical records of all pediatric patients diagnosed with unilateral intra-abdominal cryptorchidism who underwent surgical intervention at our department between July 1, 2020, and June 30, 2022. The diagnosis was confirmed preoperatively by color Doppler ultrasonography or computed tomography (14).

#### 2.1.1 Inclusion criteria

Patients were enrolled if they met the following criteria: (1) an empty scrotum on physical examination; (2) age greater than 6 months at the time of surgery; (3) unilateral intra-abdominal localization of the testis confirmed by imaging; (4) no previous surgical treatment for cryptorchidism; and (5) availability of complete clinical and follow-up data.

#### 2.1.2 Exclusion criteria

Patients were excluded for any of the following: (1) age ≤6 months; (2) non-intra-abdominal (such as canalicular) cryptorchidism; (3) anorchia or vanishing testis syndrome; (4) presence of concurrent orchitis, epididymitis, or testicular torsion; (5) recurrent cryptorchidism; or (6) history of ipsilateral scrotal or inguinal surgery. Patients with severe systemic disease contraindicating anesthesia were also excluded.

### 2.2 Group allocation and surgical techniques

Eligible patients were assigned to two groups based on the surgical procedure they underwent. The allocation was non-randomized and influenced by evolving institutional preferences and the surgeon's expertise during the study period (15). Both surgical techniques were performed concurrently throughout the study period (July 2020 to June 2022). The choice of procedure was based on evolving institutional protocols and surgeon preference, rather than a sequential transition from one technique to the other.

#### 2.2.1 Group A (modified shehata)

Underwent the two-stage modified Shehata traction orchiopexy.

#### 2.2.2 Group B(laparoscopic One-stage)

Underwent conventional laparoscopic one-stage orchiopexy. A team of experienced pediatric urologists performed all surgical procedures.

#### 2.2.3 Modified shehata surgery (group A)

##### 2.2.3.1 First stage

**Figure 1:**
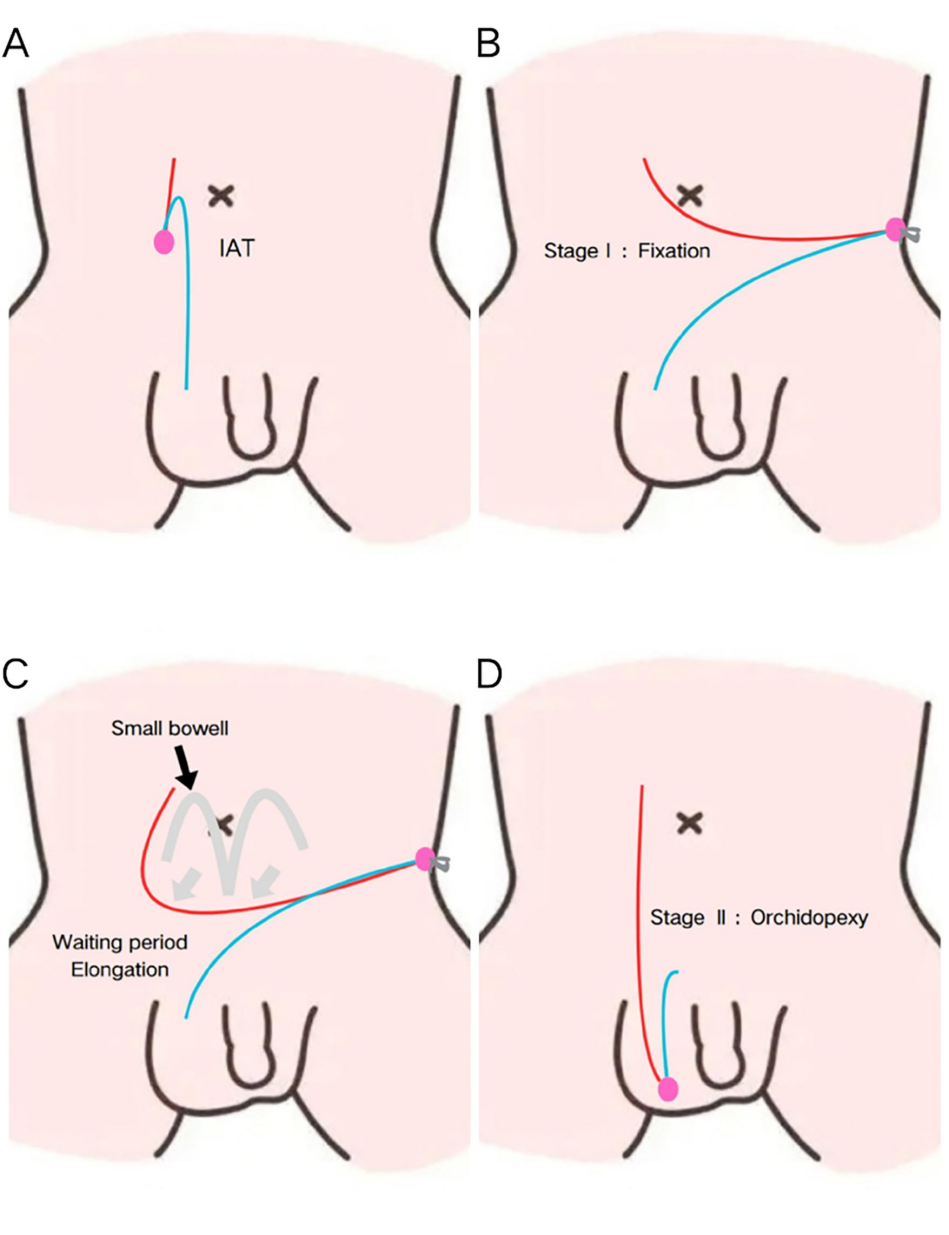
Surgical procedures of the modified shehata technique group: **(A)** intra-abdominal testis; **(B)** fixation in the first stage; **(C)** progressive elongation during the waiting period (4 weeks); **(D)** position in the scrotum in the second stage.

With the patient in the supine position, a standard laparoscopic approach was used. After identification of the intra-abdominal testis, the gubernaculum was divided using dissection forceps and electrocautery. The peritoneum overlying the spermatic cord vessels and vas deferens was carefully incised, and limited mobilization was performed. The testis was then tractioned to a position one inch superior and medial to the contralateral anterior superior iliac spine. It was fixed at this location using a 2–0 high-molecular-weight polypropylene suture (Prolene®), ensuring the testis was free of torsion and tension, with preserved vascularity. The abdominal incisions were closed (16) (Figure 1).

##### 2.2.3.2 Second stage

**Figure 2:**
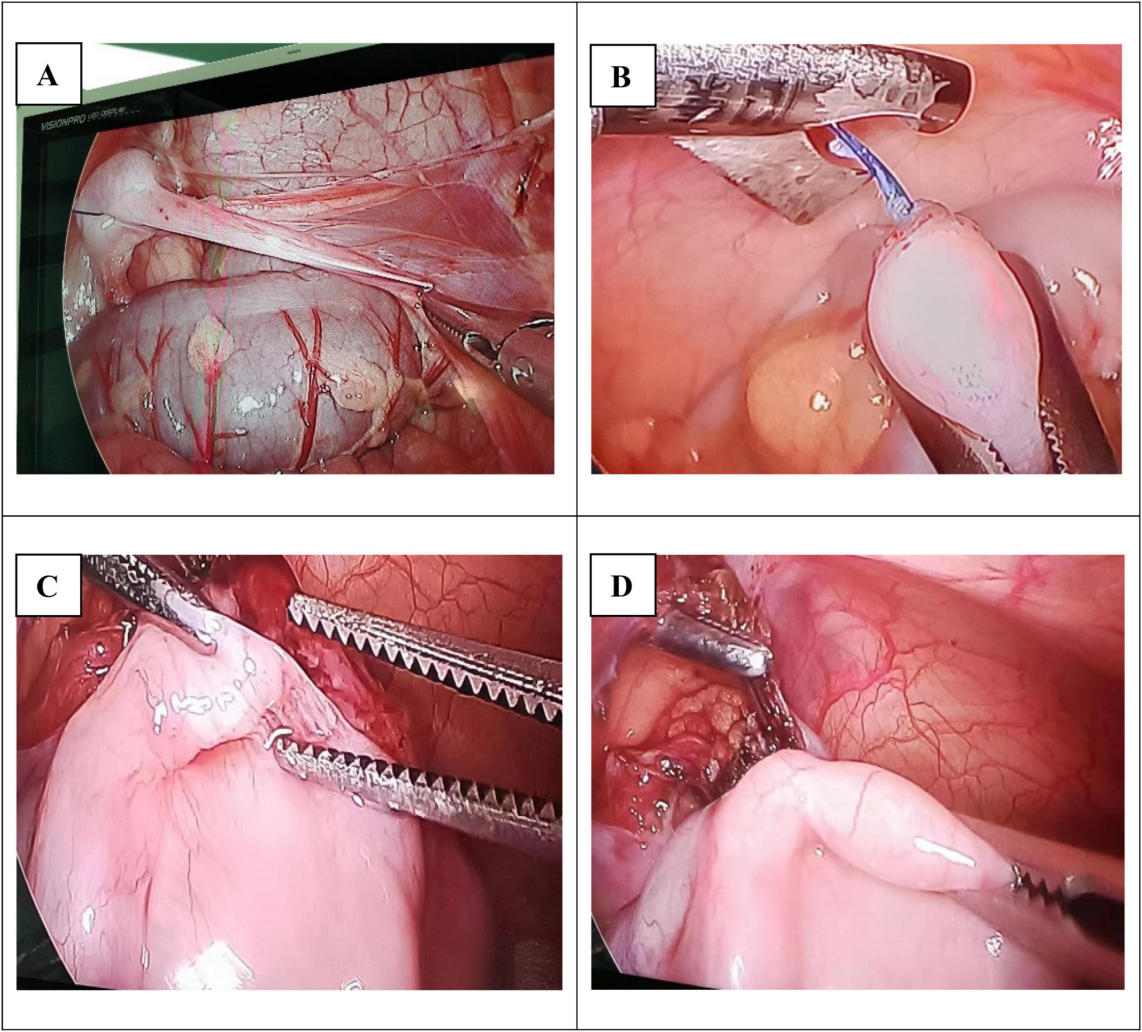
Surgical schematic diagrams: **(A)** first stage of the modified shehata procedure (4-week interval); **(B)** second stage of the modified shehata procedure. **(C,D)** Laparoscopic one-stage orchiopexy.

Four weeks after the initial surgery, a second laparoscopic procedure was performed. The abdominal cavity was re-entered to assess for adhesions, suture integrity, and the length of the testicular vessels. The fixation suture was divided, and the testis was mobilized. The achieved vessel length allowed the testis to be easily brought down to the ipsilateral internal inguinal ring. A scrotal incision was then made, a subdartos pouch was created, and the testis was transferred and secured within the pouch without tension (Figures 2A,B).

##### 2.2.3.3 Laparoscopic One-stage orchiopexy (group B)

With the patient supine, laparoscopy confirmed the intra-abdominal position of the testis. The peritoneum around the internal ring was incised, and the spermatic cord was meticulously mobilized to its maximum length while preserving the integrity of the spermatic vessels. The gubernaculum was divided. Through a scrotal incision, a subdartos pouch was created. A grasping instrument was passed from the scrotum into the abdominal cavity to grasp the testis, which was then delivered into the scrotum. The testis was fixed in the subdartos pouch, ensuring no torsion or tension on the spermatic cord (15) (Figure 2 C, D).

### 2.3 Outcome measures and efficacy evaluation

Patients were followed up at 1, 3, and 6 months postoperatively. The primary outcomes were testicular volume and blood flow. Secondary outcomes included hormonal levels and complication rates. To minimize assessment bias, the clinicians performing the ultrasonography and evaluating the images (for testicular volume and blood flow) were blinded to patients’ group allocation. The laboratory personnel conducting the hormonal assays were also blinded.

#### 2.3.1 Testicular volume and blood flow

Testicular volume (V) was measured preoperatively and at each follow-up using high-resolution ultrasonography (Toshiba Aplio 500). Volume was calculated using the ellipsoid formula: V (cm^3^) = length   ×   Width   ×   Thickness   ×   0.521 (17, 18). Color Doppler assessed testicular blood flow and semi-quantitatively categorized it as: ‘Rich’ (normal parenchymal flow), ’Slight’ (diminished but present), ‘Punctate’ (minimal spotty flow), or ‘Poor’ (absent flow) (19).

Hormonal Assays: Venous blood samples were drawn preoperatively and at the 6-month follow-up to measure serum levels of Testosterone (T), Follicle-Stimulating Hormone (FSH), and Estradiol (E2) (20, 21).

#### 2.3.2 Complications and efficacy

Early complications (such as fever, wound infection) and late complications (such as testicular retraction, atrophy) were recorded. Surgical success was strictly defined as: (1) the testis residing at the scrotal base confirmed by both physical examination and ultrasonographic localization at 6 months; (2) adequate blood flow on ultrasound with a volume reduction of <20% compared to the first postoperative measurement; and (3) absence of testicular fibrosis, atrophy (volume reduction >50%), or malignant transformation (22–24).

### 2.4 Statistical analysis

Statistical analyses were performed using SPSS software (Version 25.0, IBM Corp, USA). Continuous variables were tested for normality with the Shapiro–Wilk test. Normally distributed data were expressed as mean ± standard deviation and compared between groups using the independent samples t-test. Non-normally distributed data (such as cost) were presented as median [interquartile range] and compared using the Mann–Whitney U test. Categorical data were expressed as frequencies (percentages) and analyzed using the Chi-square test or Fisher's exact test, as appropriate. Changes in testicular volume over time (preoperative, 1, 3, and 6 months) were analyzed using a two-way repeated-measures ANOVA, with *post-hoc* Bonferroni correction for multiple comparisons. A two-tailed *P*-value of < 0.05 was considered statistically significant for all tests.

## 3 Results

### 3.1 Patient demographics and perioperative data

A total of 70 pediatric patients with unilateral intra-abdominal cryptorchidism were included in the final analysis, with 35 patients allocated to each surgical group. All procedures were completed without conversion to open surgery. The two groups were well-matched at baseline, as detailed in Table 1. There were no statistically significant differences between the Modified Shehata group and the Laparoscopic One-Stage group regarding mean age, distribution of the affected side, operative time, estimated blood loss, or duration of hospital stay (all *P* > 0.05), confirming the comparability of the cohorts for subsequent outcome analyses.

**Table 1:** Comparison of baseline and perioperative characteristics.

Characteristic	Group A (*n* = 35)	Group B (*n* = 35)	Statistical Test Value	*P*-value
Age (months), mean ± SD	23.20 ± 8.13	25.57 ± 7.69	t = 1.253	0.214
Affected Side, *n* (%)			*χ*^2^ = 0.245	0.621
-Left	12 (34.29)	14 (40.00)		
-Right	23 (65.71)	21 (60.00)		
Operative time (min), mean ± SD	89.29 ± 18.67	80.54 ± 24.73	t = 1.669	0.100
Blood Loss (mL), median [IQR]	4 [3, 5]	3 [2, 5]	Z = 1.930	0.054
Hospital Stay (days), median [IQR]	4 [3, 5]	4 [3, 6]	Z = 0.673	0.501

### 3.2 Primary outcomes

#### 3.2.1 Postoperative testicular volume

The longitudinal changes in testicular volume are summarized in Table 2. A two-way repeated-measures ANOVA revealed significant main effects for both the surgical group (F = 10.368, *P* < 0.001) and Time (F = 918.410, *P* < 0.001). A significant group-by-time interaction was also observed (F = 83.230, *P* < 0.001), indicating that the pattern of testicular growth over time differed between the two techniques.

**Table 2:** Comparison of testicular volume (cm^3^, mean ± SD) over time.

Group	Preoperative	1 Month Postop	3 Months Postop	6 Months Postop
Group A (*n* = 35)	0.50 ± 0.20	0.67 ± 0.22	0.84 ± 0.25	1.02 ± 0.25
Group B (*n* = 35)	0.47 ± 0.18	0.53 ± 0.19	0.64 ± 0.21	0.75 ± 0.21
*P*-value	0.523	0.007	< 0.001	< 0.001

Post-hoc analyses confirmed that while preoperative testicular volumes were comparable between the groups (*P* = 0.523), the Modified Shehata group exhibited significantly larger testicular volumes at the 1-month (*P* = 0.007), 3-month (*P* < 0.001), and 6-month (*P* < 0.001) follow-up intervals compared to the Laparoscopic One-Stage group.

#### 3.2.2 Testicular blood flow at 6 months

Assessment of testicular perfusion via color Doppler ultrasonography at the 6-month follow-up demonstrated a superior vascular profile in the Modified Shehata group (Table 3). A significantly higher proportion of testes in the Modified Shehata group exhibited ‘Rich’ blood flow (97.1% vs. 77.1%, *P* = 0.032). Impairments in perfusion (‘Punctate’ or ‘Poor’ flow) were observed exclusively in the Laparoscopic One-Stage group (4 cases, 11.43%).

**Table 3:** Testicular blood flow signals at 6-month follow-up, *n* (%).

Group	Rich	Slight	Punctate	Poor	*P*-value
Group A (*n* = 35)	34 (97.1%)	1 (2.9%)	0 (0.0%)	0 (0.0%)	0.032
Group B (*n* = 35)	27 (77.1%)	2 (5.7%)	4 (11.4%)	2 (5.7%)	

### 3.3 Secondary outcomes

#### 3.3.1 Hormonal outcomes

Preoperative serum levels of E2, FSH, and T were balanced between the two groups (all *P* > 0.05, Table 4). At the 6-month postoperative assessment, both groups showed a favorable hormonal shift, characterized by an increase in T and decreases in FSH and E2. However, these improvements were significantly more pronounced in the Modified Shehata group, which demonstrated a higher mean T level and lower mean FSH and E2 levels compared to the Laparoscopic One-Stage group (all *P* < 0.05, Table 4).

**Table 4:** Preoperative and 6-month postoperative hormonal profiles (mean ± SD).

Hormone	Group	Preoperative	6th Month Postoperative	*P*-value (Between Groups at 6th Month)
E2 (pmol/L)	Group A	42.17 ± 2.29	33.46 ± 2.47	<0.001
	Group B	43.04 ± 1.75	35.66 ± 1.90	
FSH (IU/L)	Group A	12.04 ± 0.24	7.70 ± 0.31	0.002
	Group B	12.12 ± 0.20	8.07 ± 0.16	
T (nmol/L)	Group A	0.36 ± 0.01	0.65 ± 0.01	<0.001
	Group B	0.35 ± 0.01	0.56 ± 0.03	

#### 3.3.2 Complications and surgical efficacy

The Modified Shehata surgery was associated with a markedly lower overall complication rate (2.86% vs. 31.43%, *P* = 0.002). As detailed in Table 5, the single complication in the Modified Shehata group was a transient postoperative fever. In contrast, the Laparoscopic One-Stage group recorded multiple complications, including 4 cases of testicular retraction and 2 cases of testicular atrophy.

**Table 5:** Postoperative complications and overall surgical efficacy.

A. Complications	Group A (*n* = 35)	Group B (*n* = 35)
Early		
-Fever	1 (2.86%)	4 (11.43%)
-Wound Infection	0	1 (2.86%)
Late		
-Testicular Retraction	0	4 (11.43%)
-Testicular Atrophy	0	2 (5.71%)
Total Complications	**1 (2.86%)**	**11 (31.43%)**
*P*-value (Total)	**0.002**	
B. Efficacy		
-Successful, *n* (%)	35 (100.0%)	29 (82.86%)
-Unsuccessful, *n* (%)	0 (0.0%)	6 (17.14%)
*P*-value	**0.033**	

Bold values indicate the total number of complications (with percentage) and the corresponding P-values for intergroup comparison.

Consequently, the Modified Shehata group achieved a 100% success rate according to the predefined efficacy criteria, which was significantly higher than the 82.86% success rate (29 of 35 patients) in the Laparoscopic One-Stage group (*P* = 0.033) (25).

#### 3.3.3 Treatment costs

A comparative analysis of total treatment costs revealed a significant economic disparity between the two approaches. The median cost for the Modified Shehata group was 17,396.22 RMB [IQR: 14,534.78–19,919.45], substantially higher than the median cost of 10,483.95 RMB [IQR: 8,986.91–11,634.70] for the Laparoscopic One-Stage group (*Z* = 6.557, *P* < 0.001).

## 4 Discussion

This retrospective study compared a modified Shehata orchiopexy with a condensed 4-week interval to laparoscopic one-stage orchiopexy for unilateral intra-abdominal cryptorchidism. The primary outcomes demonstrated the modified Shehata technique's superiority in promoting testicular volume growth and preserving robust perfusion at 6 months. Secondary outcomes, including more favorable short-term hormonal shifts, a lower rate of surgery-related complications, and a higher operative success rate, further supported the potential benefits of this staged, traction-based approach, albeit at a significantly higher total cost.

The observed superiority in primary anatomical outcomes—testicular volume and perfusion—aligns with the fundamental principle of the Shehata technique: gradual traction-induced elongation of the spermatic vessels (7). This approach minimizes acute tension on the cord during definitive placement, a key factor implicated in testicular atrophy and retraction (16, 24). In contrast, single-stage mobilization, even with meticulous technique, may occasionally stretch the vascular pedicle beyond its compensatory capacity, particularly in cases with initially borderline length. This is reflected in our findings, where impairments in blood flow and cases of atrophy occurred exclusively in the one-stage group. It is important to contextualize this comparison: while two-stage procedures are often preferred for high intra-abdominal testes, one-stage orchiopexy remains a viable and commonly attempted option when intraoperative assessment suggests adequate vessel length (4, 15). Our study, therefore, provides comparative data relevant to this specific clinical decision-making scenario.

A central finding is the validation of the 4-week inter-stage interval. This modification, pioneered at our center based on prior favorable experience (11), aims to drastically reduce the duration of intra-abdominal testicular exposure while harnessing the benefits of vascular traction. The 100% success rate and excellent perfusion outcomes suggest that sufficient vascular adaptation can occur within this abbreviated period, challenging the necessity of the conventional 8–12 week wait. This aligns with the growing interest in optimizing traction protocols to accelerate scrotal placement (12). However, a direct comparative evaluation against the standard Shehata technique with a longer interval is required to definitively establish the superiority of this modified timeline.

The secondary outcomes related to hormonal profiles warrant careful and cautious interpretation. In prepubertal boys, the hypothalamic-pituitary-gonadal axis is quiescent, and serum sex hormone levels are low and dynamic, influenced by factors such as the transient activation during “mini-puberty” (26–28). The statistically significant differences observed at 6 months (higher T, lower FSH/E2 in the modified Shehata group) are intriguing and correlate with better anatomical outcomes. However, they should not be overinterpreted as definitive evidence of superior Leydig or Sertoli cell function at this early stage. These changes may reflect differences in the rate of testicular microenvironment recovery, the resolution of surgical stress, or variations in the timing of individual endocrine rhythms. They are presented as preliminary, exploratory data that complement the primary anatomical endpoints. Robust assessment of endocrine function and fertility potential necessitates long-term follow-up into and beyond puberty (21, 29).

The significantly lower surgery-related complication rate (atrophy and retraction) and consequent higher success rate in the modified Shehata group underscore a key clinical advantage. The higher overall complication rate reported in Table 5 for Group B is influenced by the inclusion of postoperative fever, a common pediatric surgical event less directly tied to surgical technique. When focusing on complications specific to orchiopexy (atrophy, retraction), the advantage of the staged, low-tension approach becomes clearer. Nevertheless, the higher economic cost of the two-stage procedure is a substantial practical consideration, reflecting the resources required for two separate anesthetic and laparoscopic operations. A formal cost-effectiveness analysis incorporating long-term outcomes and costs of managing complications would be valuable.

This study has the following limitations. A retrospective, non-randomized design introduced the possibility of selection bias, although baseline demographics were comparable. Crucially, the detailed anatomical data that usually guide the choice of surgery (e.g., the precise position of the testis relative to the inner ring, the initial length of the spermatic vessels) are not recorded in detail, limiting our ability to confirm the anatomical equivalence of each group at baseline. The single-center experience and modest sample size affect generalizability. The 6-month follow-up, while adequate for assessing primary anatomical outcomes, is too short to evaluate long-term fertility. Furthermore, the postoperative assessment protocol involving serial ultrasonography and hormone assays, while part of our institutional practice for monitoring these cases, is not universally standardized. Finally, this study compared the modified Shehata technique specifically to one-stage orchiopexy. While this addresses a relevant clinical question, it does not establish its superiority over other established two-stage techniques, such as the standard Shehata or Fowler-Stephens procedures. Future research should involve prospective, randomized trials with long-term follow-up, direct comparison to other staged techniques, and careful documentation of pre-operative anatomical parameters.

## 5 Conclusion

This retrospective study compared two surgical approaches—a modified two-stage Shehata technique with a 4-week interval and laparoscopic one-stage orchiopexy—for the management of unilateral intra-abdominal cryptorchidism. Within the context of this specific comparison, the modified Shehata procedure was associated with more favorable outcomes at 6 months postoperatively, including larger testicular volume, better-preserved blood flow, lower surgery-related complication rates, and a higher success rate, although at a higher total cost. The findings suggest that shortening the inter-stage interval to 4 weeks may be a feasible modification to the original Shehata technique.

It is important to acknowledge, however, that neither of the surgical techniques evaluated in this study is currently established as a guideline-endorsed standard of care for intra-abdominal cryptorchidism. The optimal surgical approach for this condition remains a subject of ongoing debate, with multiple valid options—including one-stage orchiopexy, Fowler-Stephens procedures, and various staged techniques—each carrying distinct advantages and limitations. Therefore, while the modified Shehata technique appears to offer certain benefits compared specifically to one-stage orchiopexy in this patient cohort, its role relative to other established surgical strategies remains to be determined. Long-term follow-up studies with larger sample sizes and direct comparisons against alternative two-stage approaches are warranted to fully assess functional outcomes, including fertility potential and endocrine function.

## Data Availability

The original datasets generated and analyzed during this study are not publicly available due to patient privacy regulations, but are available from the corresponding author upon reasonable request.
